# 

**Published:** 1910-08

**Authors:** 


PUBLISHED MONTHLY. ATLANTA SUBSCRIPTION $1.00

### JOURNAL-RECORD OF MEDICINE

LIBRARY
Indexed

Successor to { **Atlanta Medical and Surgical Journal, Established 1855.**

**and Southern Medical Record, Established 1870.** } **56th Year**

Vol. LVI. ATLANTA, GA., AUGUST, 1910. No. 5

